# Long-term bedside dynamic monitoring of jejunal mucosa via an indwelling visualized nasojejunal tube in a neurocritical patient: a case report and proposal for a novel scoring system

**DOI:** 10.3389/fnut.2026.1802293

**Published:** 2026-04-01

**Authors:** Xiaoyun Li, Hongtang Liang, Yongfu Zheng

**Affiliations:** 1 Department of Critical Care Medicine, The First Affiliated Hospital, Sun Yat-sen University, Guangzhou, China; 2 Guangdong Clinical Research Center for Critical Care Medicine, Guangzhou, China

**Keywords:** case report, enteral nutrition, jejunal mucosa, nasojejunal tube, visualized

## Abstract

**Background:**

Enteral nutrition (EN) intolerance is a prevalent challenge in neurocritical care. Traditional markers, such as gastric residual volume, remain controversial due to their low specificity. While visualized nasojejunal tubes (NJT) facilitate placement, their application as an indwelling monitoring tool for observing real-time mucosal dynamics remains unexplored.

**Case description:**

We report a case of a 62-year-old male with cerebral hemorrhage who required post-pyloric feeding due to high aspiration risk and initial gastric feeding intolerance. An indwelling hydrophilic-coated visual NJT with a distal tip camera was deployed. Serial bedside monitoring was performed on Days 1, 7, 14, and 21 over a 22-day indwelling period. We established a novel “Bedside Visual Mucosal Scoring System” to semi-quantitatively assess mucosal color, villi morphology, and luminal contents. Serial monitoring revealed a distinct transition from an initial edematous and hyper-secretory state to a healthy state with distinct villi architecture. Notably, the visual improvement of mucosal integrity coincided chronologically with the patient’s clinical stabilization, including decreased inflammatory markers and successful weaning from mechanical ventilation. The patient achieved full caloric targets without catheter-related complications.

**Conclusion:**

This case demonstrates the feasibility and safety of using hydrophilic-coated visual NJT for long-term, bedside dynamic monitoring of jejunal mucosa. This technique offers a promising alternative from indirect assessment to direct visualization, highlighting the potential for mucosa-directed precision nutrition strategies in critically ill patients.

## 1 Introduction

Critically ill patients with stroke frequently suffer from gastrointestinal dysfunction and enteral nutrition (EN) intolerance, which are associated with increased infection rates and mortality (1–3). Although early EN is recommended, current clinical practice relies on indirect indicators like gastric residual volume (GRV), which lack sensitivity and fail to provide early warning for precision intervention (4). Furthermore, existing assessment methods—such as biochemical markers or standard endoscopy—are limited by low specificity, discontinuity, or invasiveness, making them unsuitable for frequent bedside monitoring in the intensive care unit (ICU) (5).

Recently, visualized nasojejunal tube (NJT) technology has emerged as a potential solution. However, previous applications were predominantly limited to placement navigation, lacking the capability for post-placement observation. In contrast, the novel device used in this case integrates an embedded camera that remains *in situ*, enabling long-term serial monitoring of the jejunal mucosa. This case report demonstrates the systematic application of a hydrophilic-coated visualized NJT for bedside mucosal monitoring in a neurocritical patient. We explore the clinical significance of dynamic morphological changes based on video observation, aiming to provide a novel perspective for the direct assessment of feeding tolerance.

## 2 Case description

A 62-year-old male with a history of hypertension presented to the emergency department in October 2025 with sudden onset headache and loss of consciousness for one hour. Upon admission, the patient was in a coma (Glasgow Coma Scale score: 6) with equal but sluggish pupils (1.5 mm). Vital signs indicated severe hypertension (189/94 mmHg, 1 kPa = 7.5 mmHg) and bradycardia (60 bpm), prompting immediate endotracheal intubation and mechanical ventilation. An emergent head CT revealed a right basal ganglia hemorrhage with intraventricular extension (involving bilateral lateral, third, and fourth ventricles). During transfer, the patient developed signs of herniation (anisocoria: right pupil 2 mm, left 1.5 mm). Emergency bilateral lateral ventricular drainage and right thalamic hematoma drainage were performed. Intraoperative hemodynamic stability was maintained with norepinephrine (0.1 μg/kg/min).

Postoperatively, the patient was admitted to the ICU. Laboratory results showed leukocytosis (white blood cell count 20.57 × 10^9^/L [reference range: 4–10 × 10^9^/L]), elevated inflammatory markers (C-reactive protein 47.12 mg/L [<10 mg/L], procalcitonin 0.21 ng/mL [<0.05 ng/mL]), and hypoalbuminemia (albumin 31 g/L [35–52 g/L], pre-albumin 98 mg/L [200–400 mg/L]). The illness severity was high, with an acute physiology and chronic health evaluation II score of 32 and a sequential organ failure assessment score of 10. Neurocritical care was managed adhering to the GHOST-CAP protocol to minimize secondary brain injury.

With an NRS-2002 score of 4 indicating high nutritional risk, early EN via a nasogastric tube was initiated on ICU Day 2 once hemodynamic stability was achieved. A polymeric formula (Nutrison, Nutricia) was administered via a continuous pump at an initial rate of 20 mL/h. Standard aspiration precautions were implemented, including head-of-bed elevation, administration of prokinetic agents (metoclopramide), and routine monitoring of GRV. Following institutional protocols based on ESPEN guidelines, we considered a GRV > 500 mL as a threshold for intolerance. Although the pre-aspiration GRV was 460 mL, on Day 3, as the infusion rate was titrated up to 40 mL/h, the patient developed hiccups followed by a regurgitation event. Enteral feed-like secretions were suctioned primarily from the oral cavity and the subglottic space above the endotracheal tube cuff. Attributable to the effective barrier provided by the endotracheal cuff and timely suctioning by the nursing team, the patient did not experience significant desaturation or immediate signs of worsening aspiration pneumonia.

**Figure 1:**
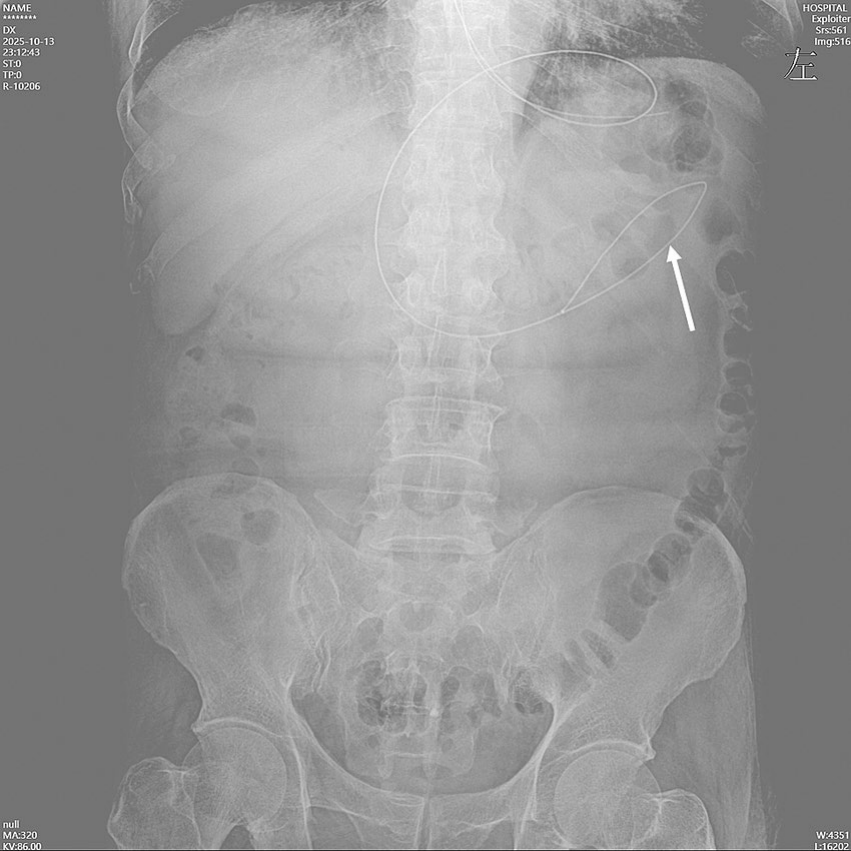
Radiographic confirmation of catheter placement. Abdominal X-ray showing the radiopaque hydrophilic-coated visualized nasojejunal tube passing through the stomach into the small intestine, with the distal tip located in the proximal jejunum (white arrow), confirming successful post-pyloric placement.

Given the evident gastric intolerance and high risk of recurrent aspiration, a decision was made to switch to post-pyloric feeding. A disposable hydrophilic-coated visualized NJT (feeding type, 8Fr, Well Lead Medical Co., Ltd., China) was inserted at the bedside with the patient in the right lateral decubitus position. Under real-time video navigation, anatomical landmarks including the gastric antrum, pylorus, and duodenal circular folds were clearly identified. The catheter tip was precisely advanced into the upper jejunum to a depth of 110 centimeters. The procedure was completed successfully in one attempt within 30 min. The final tip position was confirmed by abdominal X-ray (Figure 1).

Following successful NJT placement, EN was restarted. To manage the risk of stress hyperglycemia, a regimen of enteral nutritional suspension (TPF-D) (Trade name: Yilijia; Abbott Laboratories B. V., Netherlands) at 10 mL/h was initiated. To assess mucosal tolerance, feeding was paused on Days 1, 7, 14, and 21. After flushing the lumen with 20 mL of 0.9% saline, 10–30 min of video showing jejunal mucosal rest and peristalsis were recorded. All video files were anonymized and stored for subsequent analysis.

**Figure 2:**
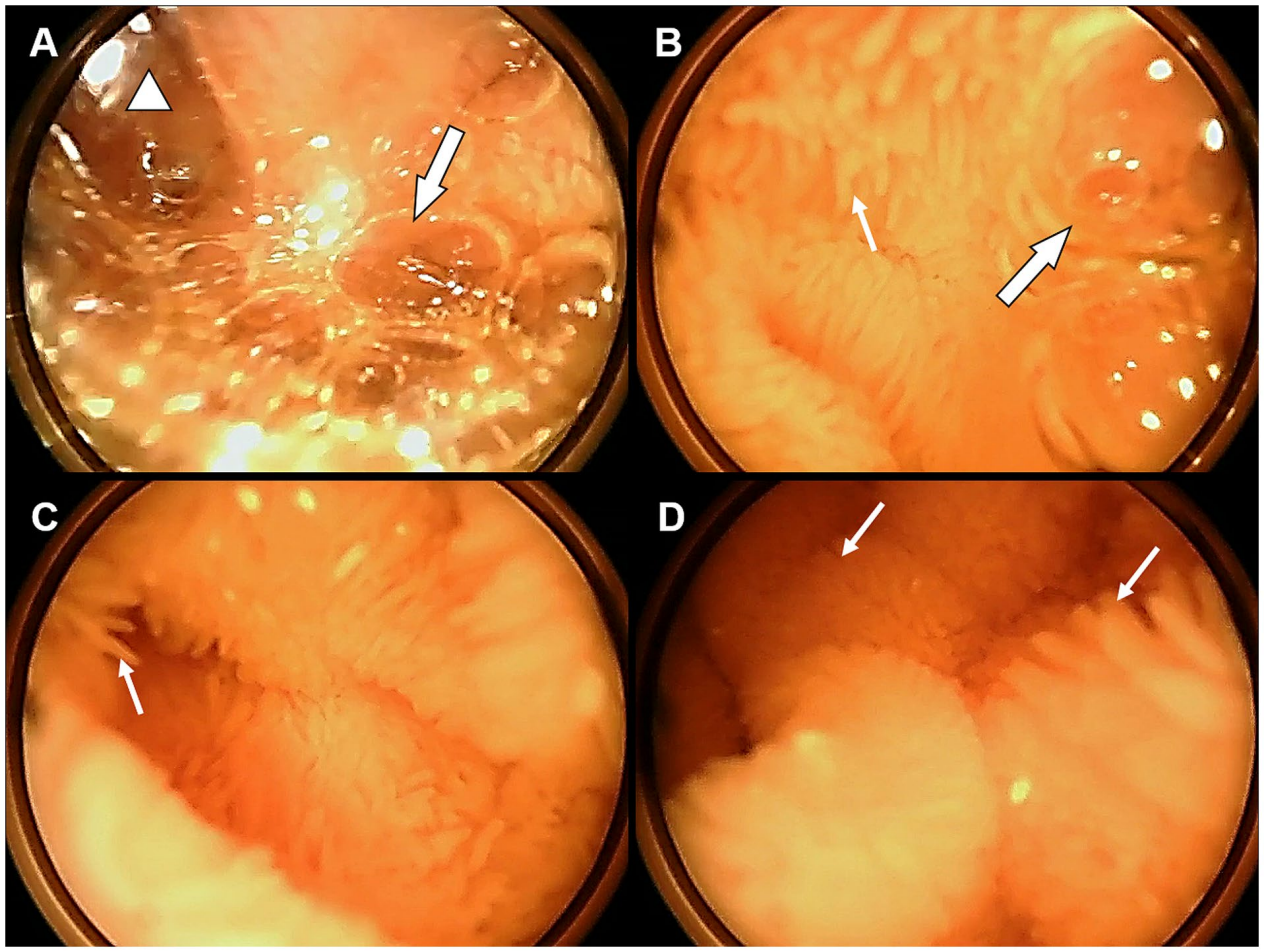
Dynamic evolution of jejunal mucosal morphology under bedside visualization. **(A)** Day 1: The lumen exhibits a hyper-secretory state with accumulation of excessive mucus (white triangle) and foamy secretions/air bubbles (thick white arrow). The mucosal surface appears edematous with strong light reflection, and villi structures are blurred. **(B)** Day 7: A reduction in luminal contents is observed. Scattered bubbles (thick white arrow) persist, but mucosal edema begins to subside. **(C)** Day 14 and **(D)** Day 21: The mucosa presents a healthy ruddy appearance with a clean lumen. Distinct, individual finger-like villi structures (thin white arrows) are clearly visible and well-arranged. No signs of mucosal pressure injury, erosion, or congestion were observed.

Serial video monitoring captured a distinct morphological transition from stress-induced alteration to functional recovery (Figure 2). Initially (Day 1), the jejunal lumen was characterized by the accumulation of excessive mucus and air bubbles, with villi structures only vaguely discernible, indicating a state of hyper-secretion and hypomotility. By Day 7, following nasogastric tube removal and EN titration, the mucosa appeared pale pink with clearly distinguishable villi, marking the early recovery from severe post-operative stress. As EN stabilized on Day 14, the mucosa presented a ruddy appearance with improved villi clarity and significantly active peristalsis; this microscopic improvement coincided chronologically with the patient’s clinical stabilization, which was characterized by declining inflammatory markers and the initiation of weaning from mechanical ventilation. Finally, prior to ICU discharge on Day 21, the mucosa maintained a healthy morphology with normal coloration and regular peristalsis. No signs of congestion, edema, or erosion were observed, confirming that mucosal integrity was well-preserved despite long-term catheter indwelling and continuous TPF-D feeding.

Using the four valid video sequences, we applied our novel “Bedside Visual Mucosal Scoring System” (Table 1) for semi-quantitative assessment. The total score ranges from 0 (normal) to 9 (severe dysfunction). This scale was developed based on the modified Mayo score (for ulcerative colitis) and capsule endoscopy scoring systems, adapted for the specific imaging characteristics of this device. Two researchers independently scored the videos (blinded to the specific timepoints). Out of 12 total scoring items, initial agreement was reached on 11 items. Statistical analysis demonstrated substantial inter-rater reliability, with a linear weighted Cohen’s kappa (*κ*) of 0.80 (95% CI: 0.43–1.00). Any discrepancies were resolved by a third adjudicator. The total composite scores showed a progressive improvement: Day 1 (Score: 3), Day 7 (Score: 1), Day 14 (Score: 0), and Day 21 (Score: 0). Based on the visual confirmation of reduced edema and clear villi on Day 7, the clinical team decided to escalate the infusion rate from 20 mL/h to 40 mL/h and subsequently to 70 mL/h, despite the patient’s history of aspiration.

**Table 1:** Bedside visual mucosal scoring system.

Items	**0**	**1**	**2**	**3**
Mucosal color	Pink (Normal)	Pale/Hyperemic (<1/3)	Diffuse Pale/Red (≥1/3)	Cyanotic/Bleeding/Purplish
Villi morphology	Distinct/Finger-like	Blurry/Edematous	Flattened/Smooth	Mucosal defect/Ulceration
Luminal contents	Clear/Little mucus	Turbid bile/Mucus	Stasis/Granular residue	Purulent/Hematic/Coffee-ground

**Figure 3:**
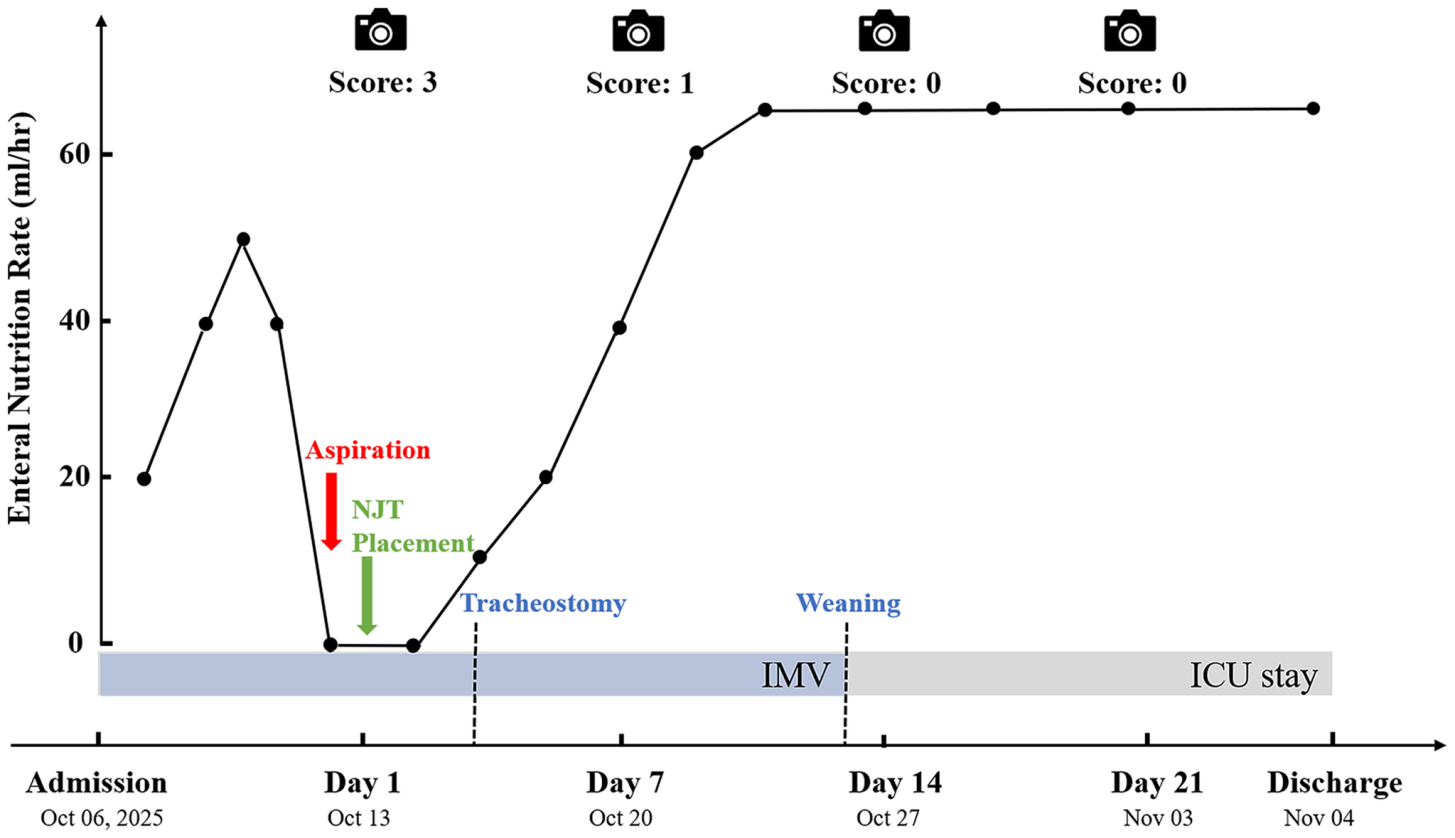
Timeline overview of clinical course, enteral nutrition trajectory, and mucosal scores. The line graph depicts the trajectory of the enteral nutrition infusion rate (ml/h). Key clinical events, including aspiration, nasojejunal tube placement, and airway management, are annotated on the timeline. Camera icons indicate the specific timing of bedside visualization with corresponding mucosal scores. Total possible score ranges from 0 (normal) to 9 (severe dysfunction). Lower scores indicate better mucosal status. The bottom bars represent the duration of invasive mechanical ventilation and ICU length of stay.

Throughout the 22-day monitoring period, no further episodes of aspiration or ventilator-associated pneumonia occurred. EN was well-tolerated, with a stable target rate of 70 mL/h (1700 kcal/day) (Figure 3). Defecation was regular (every 2–4 days), with yellowish-brown soft stool (Bristol type 6). The patient showed gradual neurological improvement and was successfully weaned from ventilation. Pre-discharge labs indicated significant nutritional and inflammatory recovery (albumin 34 g/L, pre-albumin 221 mg/L, C-reactive protein 9.76 mg/L). The patient was transferred to a rehabilitation facility on day 29. No catheter-related complications, such as blockage, displacement, mucosal pressure injury, or local infection, were observed.

## 3 Discussion

To our knowledge, this is among the first reported cases describing the use of a hydrophilic-coated visualized NJT extending from traditional placement navigation to dynamic mucosal monitoring during the feeding period. Through serial observation over a 22-day indwelling period, we demonstrated the feasibility of performing bedside, non-invasive, and dynamic visualization of the jejunal mucosa in a critically ill patient. This practice transcends the limitations of relying solely on indirect indicators, such as GRV, and establishes a preliminary framework based on the “Bedside Visual Mucosal Scoring System,” offering a novel mucosa-directed perspective for evaluating EN tolerance.

The intestinal mucosa serves as a vital physiological barrier composed of tight junctions (mechanical barrier) and gut-associated lymphoid tissue (immune barrier) (2, 6). In critical illness, systemic inflammation and hemodynamic instability can severely compromise this barrier, leading to increased epithelial apoptosis, loss of tight junctions, and subsequent hyperpermeability. This disruption increases the risk of bacterial translocation, which may drive systemic inflammatory response syndrome and multiple organ dysfunction (6, 7).

EN plays an irreplaceable role in maintaining this barrier by directly nourishing epithelial cells, stimulating peristalsis, and promoting immune defense (2, 8). Multiple studies have confirmed that early EN attenuates the inflammatory response, reduces intestinal permeability, stabilizes the gut microbiota, promotes barrier recovery, and improves clinical outcomes (7, 9–12). In our case, the continuous post-pyloric feeding via the visualized NJT coincided chronologically with favorable mucosal scores and the absence of infectious complications. This clinical observation visually corroborates the protective mechanism of EN, suggesting that visible mucosal recovery may reflect the restoration of barrier function.

Despite the critical importance of EN, clinical practice lacks effective, real-time, and intuitive tools to assess its true impact on the mucosa. Current assessment methods have significant limitations. Clinical indicators such as GRV, intra-abdominal pressure, and defecation status are non-specific and lagging (4). Serum biomarkers like plasma citrulline, intestinal fatty acid-binding protein, or diamine oxidase, while reflective of damage, are confounded by systemic metabolism, lack localization, and suffer from time delays (2, 13). Imaging modalities fail to visualize microscopic morphology. Standard endoscopy, while the gold standard, is too invasive and logistically impractical for frequent bedside monitoring in the ICU (11). Consequently, the lack of a clear definition for EN intolerance and effective bedside monitoring methods often leads to nutrition management based on empirical judgment rather than objective evidence.

The visualized NJT technology employed in this case bridges this diagnostic gap. Its core advantage lies in enabling bedside, serial, and direct monitoring. Unlike the blind management inherent to traditional tubes, this technology allows for daily activation of the camera to directly observe the mucosa along the feeding path. In this case, we captured the structural transition from an edematous state to one with distinct villi, providing objective visual evidence to support the titration of the nutrition regimen to full caloric targets. This visualization capability allows clinicians to distinguish whether intolerance stems from etiologies such as hypoperfusion or local inflammation, enabling more precise, individualized intervention.

Safety is a primary concern for long-term indwelling devices. Critical illness is characterized by splanchnic hypoperfusion, making the mucosa susceptible to pressure necrosis. In our case, despite 22 days of retention, no mucosal pressure injuries or erosions were observed. We hypothesize that this favorable safety profile may be attributable to two factors: First, the early initiation of EN directly nourished the epithelial cells and improved local microcirculation. Second, the hydrophilic coating of the catheter, upon hydration, significantly reduced the coefficient of friction between the catheter and the intestinal wall during peristalsis. This combined effect of advanced material science and early physiological stimulation may explain the favorable safety profile observed in this case, supporting its potential as a long-term monitoring tool.

The implementation of this technology requires consideration of cost and training. The device costs approximately 217 USD (1,500 RMB). While the unit cost exceeds that of standard nasojejunal tubes (129 USD / 890 RMB), it offers potential long-term cost-effectiveness. Specifically, the visualized tube is approved for an extended indwelling time of up to 60 days (vs. 30 days for standard tubes), which reduces the frequency of replacements and the need for repeated radiographic confirmations. Regarding implementation, the research team completed accredited National/Provincial-level training programs (comprehensive 3- to 5-day courses). This standardized training is essential to ensure safe placement and objective image interpretation. Furthermore, the device is single-use and sterile, eliminating complex reprocessing protocols and minimizing cross-infection risks. To prevent thermal mucosal injury, camera activation is limited to intermittent monitoring (≤30 min per session).

As a preliminary exploration, this report has limitations. First, the safety profile observed in this single case cannot be generalized; comparative studies are needed to rule out potential pressure injuries in a larger cohort. Second, the observation range is limited to the proximal jejunum, and whether this represents the functional status of the entire small intestine remains to be verified. Furthermore, while the current scoring system demonstrated good inter-rater reliability, it remains semi-quantitative and requires validation against histopathology or biomarkers. Nevertheless, this case successfully demonstrates the potential for critical care nutrition management to advance toward more precise, visual mucosa-level precision medicine.

## 4 Conclusion

This case report successfully demonstrates the feasibility and safety of utilizing hydrophilic-coated visualized NJT for long-term, serial bedside monitoring of jejunal mucosa in critically ill patients. Surpassing traditional indirect assessment methods, this technology provides a direct and real-time visual perspective for evaluating enteral nutrition tolerance. Although large-scale studies are warranted to establish a standardized assessment system, this work marks a critical step toward more precise, personalized mucosa-level monitoring in critical care nutrition, holding significant clinical and research implications.

## Data Availability

The datasets presented in this article are not readily available because of ethical and privacy restrictions. Requests to access the datasets should be directed to the corresponding author/s.

## References

[1] Chen R Wan J Li J Tu Y. The effect of cluster nursing combined with early enteral nutrition intervention in postoperative severe craniocerebral injury. Langenbeck's Arch Surg. (2025) 410:148. doi: 10.1007/s00423-025-03699-4, 40293452 PMC12037423

[2] Li P Jian JN Chen RL. Effect of early enteral nutrition on serum inflammatory factors and intestinal mucosal permeability in patients with severe acute pancreatitis. Turk J Gastroenterol. (2021) 32:907–12. doi: 10.5152/tjg.2021.201033, 34787096 PMC8975450

[3] Lopez-Gomez JJ Delgado Garcia E Primo-Martin D de la Simon Fuente M Gomez-Hoyos E Jimenez-Sahagun R . Effect of a diabetes-specific formula in non-diabetic inpatients with stroke: a randomized controlled trial. Nutr Diabetes. (2024) 14:34. doi: 10.1038/s41387-024-00292-4, 38816400 PMC11139872

[4] Meng M Klingensmith NJ Coopersmith CM. New insights into the gut as the driver of critical illness and organ failure. Curr Opin Crit Care. (2017) 23:143–8. doi: 10.1097/mcc.0000000000000386, 28092310 PMC5373099

[5] Nunes G Guimaraes M Coelho H Carregosa R Oliveira C Pereira SS . Prolonged fasting induces histological and ultrastructural changes in the intestinal mucosa that may reduce absorption and revert after enteral refeeding. Nutrients. (2023) 16:128. doi: 10.3390/nu16010128, 38201958 PMC10780540

[6] Oami T Shimazui T Yumoto T Otani S Hayashi Y Coopersmith CM. Gut integrity in intensive care: alterations in host permeability and the microbiome as potential therapeutic targets. J Intensive Care. (2025) 13:16. doi: 10.1186/s40560-025-00786-y, 40098052 PMC11916345

[7] Oami T Yamamoto A Ishida S Kondo K Hata N Oshima T. Critical care nutrition from a metabolic point of view: a narrative review. Nutrients. (2025) 17:1352. doi: 10.3390/nu17081352, 40284216 PMC12029973

[8] Patel JJ Barash M. The gut in critical illness. Curr Gastroenterol Rep. (2025) 27:11. doi: 10.1007/s11894-024-00954-4, 39792234

[9] Quiroz-Olguin G Gutierrez-Salmean G Posadas-Calleja JG Padilla-Rubio MF Serralde-Zuniga AE. The effect of enteral stimulation on the immune response of the intestinal mucosa and its application in nutritional support. Eur J Clin Nutr. (2021) 75:1533–9. doi: 10.1038/s41430-021-00877-7, 33608653

[10] Reignier J Gaillard-Le Roux B Dequin PF Bertoni Maluf VA Bohe J Casaer MP . Expert consensus-based clinical practice guidelines for nutritional support in the intensive care unit: the French Intensive Care Society (SRLF) and the French-speaking Group of Pediatric Emergency Physicians and Intensivists (GFRUP). Ann Intensive Care. (2025) 15:99. doi: 10.1186/s13613-025-01509-0, 40665004 PMC12263543

[11] Singer P Blaser AR Berger MM Calder PC Casaer M Hiesmayr M . ESPEN practical and partially revised guideline: clinical nutrition in the intensive care unit. Clin Nutr. (2023) 42:1671–89. doi: 10.1016/j.clnu.2023.07.011, 37517372

[12] Sluis WM de Jonge JC Reinink H Woodhouse LJ Westendorp WF Bath PM . Metoclopramide to prevent pneumonia in patients with stroke and a nasogastric tube: data from the PRECIOUS trial. Stroke. (2024) 55:2402–8. doi: 10.1161/STROKEAHA.124.047582, 39129650 PMC11419274

[13] Zhao J Yuan F Song C Yin R Chang M Zhang W . Safety and efficacy of three enteral feeding strategies in patients with severe stroke in China (OPENS): a multicentre, prospective, randomised, open-label, blinded-endpoint trial. Lancet Neurol. (2022) 21:319–28. doi: 10.1016/S1474-4422(22)00010-2, 35219379

